# Comprehensive analysis of pyroptosis regulators and tumor immune microenvironment in clear cell renal cell carcinoma

**DOI:** 10.1186/s12935-021-02384-y

**Published:** 2021-12-14

**Authors:** Yan Zhang, Xianwu Chen, Qinghe Fu, Feifan Wang, Xuejian Zhou, Jiayong Xiang, Ning He, Zhenghui Hu, Xiaodong Jin

**Affiliations:** Department of Urology, The First Affiliated Hospital, Zhejiang University School of Medicine, Hangzhou, 310003 Zhejiang People’s Republic of China

**Keywords:** Clear cell renal cell carcinoma, Pyroptosis, Tumor immune microenvironment, Immune checkpoints, Immunotherapy

## Abstract

**Background:**

Increasing evidence has indicated that pyroptosis could regulate the tumor immune microenvironment (TIME) to affect the tumor development. As a highly immunogenic tumor, clear cell renal cell carcinoma (ccRCC) can benefit from immunotherapy, but related research on pyroptosis in the TIME of ccRCC is still deficient.

**Methods:**

Available data derived from TCGA and GEO databases were analyzed to identify the different expression profiles of pyroptosis in ccRCC and normal tissues, and the correlation of pyroptosis regulators with TIME was evaluated in ccRCC.

**Results:**

According to consensus clustering analysis, two differential expression levels of subtypes were identified to affect patient prognosis, and were related to histological tumor stage and grade. Immune cells were calculated by the CIBERSORT algorithm. Higher infiltrated levels of B cells naive, T cells CD4 memory resting, NK cells resting, monocytes, macrophages were observed in Cluster 1, while higher infiltrated levels of CD8^+^ T cells, T follicular helper cells, and Tregs were observed in Cluster 2. Gene set enrichment analysis indicated that Cluster 2 was enriched in multiple immune-related pathways, including the JAK-STAT signaling pathway. Moreover, overexpression of eight immune checkpoints was related to ccRCC development, especially in Cluster 2. As four potentially key pyroptosis regulators, AIM2, CASP5, NOD2, and GZMB were confirmed to be upregulated in ccRCC by RT-qPCR analysis and further verified by the HPA database. Further pan-cancer analysis suggested that these four pyroptosis regulators were differentially expressed and related to the TIME in multiple cancers.

**Conclusion:**

The present study provided a comprehensive view of pyroptosis regulators in the TIME of ccRCC, which may provide potential value for immunotherapy.

**Supplementary Information:**

The online version contains supplementary material available at 10.1186/s12935-021-02384-y.

## Background

Renal cell carcinoma (RCC) is the most common malignant solid tumor of the kidney in adults, accounting for approximately 3% of all adult malignancies and 90% of renal malignancies [1]. At present, the number of deaths due to RCC each year worldwide has exceeded 1,00,000, and the morbidity and mortality rates are increasing at a rate of 2–3% every 10 years [2]. According to the 2004 version of WHO’s RCC pathological classification, the incidence of clear cell renal cell carcinoma (ccRCC) is approximately 70% [3]. Although surgical resection is still the best treatment for ccRCC, there is currently no effective postoperative adjuvant treatment, and ccRCC is not sensitive to radiotherapy and chemotherapy. According to the literature, 20–40% of patients experience recurrence after surgery [4]. As a highly immunogenic tumor, ccRCC can benefit from immunotherapy.

Pyroptosis is a type of programmed cell death accompanied by inflammation triggered by pathogenic microorganism infection or other harmful signals [5]. Pyroptosis is mainly characterized by cell swelling, lysis and release of cytoplasmic contents. Appropriate pyroptosis is an important mechanism for the host to resist infection by foreign pathogenic microorganisms. However, excessive pyroptosis is harmful or even fatal to the host. An increasing number of studies have shown that pyroptosis also plays an important role in the development of tumors [6]. Pyroptosis creates a tumor-inhibiting environment by releasing inflammatory factors. However, pyroptosis can also weaken the body’s immune function to tumor cells and accelerate the growth of tumors in different cancers. Some studies have shown that the effective proinflammatory effect of pyroptosis is related to the regulation of the tumor immune microenvironment [7]. These findings suggest that pyroptosis plays an important role in tumor development and anti-tumor process. However, the effect of pyroptosis on the prognosis of ccRCC is unclear. A comprehensive understanding of pyroptosis in ccRCC is still lacking, including the crosstalk between pyroptosis regulators and the tumor immune microenvironment.

In the present study, we performed a comprehensive retrospective analysis based on The Cancer Genome Atlas (TCGA) database and the Gene Expression Omnibus Database (GEO) to estimate the influence of pyroptosis on the TIME, and further explored the underlying mechanisms between pyroptosis and individual TIME characterizations based on the consensus cluster analysis. In addition, the present study also verified the relationship between pyroptosis and immune hot checkpoints expression.

## Materials and methods

### Data acquisition and identification of differential expression

The RNA sequencing (RNA-seq) data of 539 ccRCC tissues and 72 normal kidney tissues as well as the corresponding clinical features from TCGA database (https://portal.gdc.cancer.gov/) were obtained. The GSE46699 and GSE53757 data from the GEO database (https://www.ncbi.nlm.nih.gov/geo/) were collected and used to evaluate pyroptosis regulator expression. Moreover, TCGA pan-cancer gene expression data for 33 types of malignant tumors and corresponding clinical information, such as stemness scores based on mRNA (RNAss) and DNA-methylation (DNAss) as well as immune subtypes, were obtained from the University California Santa Cruz database (UCSC Xena, http://xena.ucsc.edu/). The 37 pyroptosis regulators are presented in Additional file 4: Table S1 and were extracted from prior investigations [8, 9]. By using the “limma” package, more than two fold changes and false discovery rate (FDR, adjusted *p *value)  < 0.05 is considered as differential gene expression. The immunohistochemistry (IHC) staining images of pyroptosis regulators in normal kidney and tumor tissues were analyzed and obtained from the HPA database (http://www.proteinatlas.org/). In order to compare the expression between normal and tumor samples, all tissues were selected that used the same antibodies.

### Consensus clustering

Univariate Cox regression analysis was used to extract pyroptosis regulators via the R package “survival” (*p* value  < 0.05). Consensus clustering was performed to explore the potential molecular subtype between the BCa patients using the R package “Consensus Cluster Plus”. The cluster count (k) was set from 1 to 9, and the best optimal k value was selected for further investigation.

### Estimation of tumor immune microenvironment

CIBERSORT, an analysis tool based on the LM22 immune gene signature, was used to evaluate the tumor immune infiltration levels of ccRCC. The algorithm was run for 1000 permutations and ccRCC samples with an output P  < 0.05 were selected for further analysis. The “ESTIMATE” package in R was used to evaluate the immune score and the stromal score. Moreover, 8 common immune checkpoint genes, including PD-1, PD-L1, PD-L2, CTLA4, SIGLEC15, TIGIT, TIM3 and LAD3, were extracted and evaluated for their correlations with pyroptosis regulators in ccRCC.

### Functional enrichment analysis

Gene Ontology (GO) analysis, and Kyoto Encyclopedia of Genes and Genomes (KEGG) signaling pathway analysis were performed using the “cluster Profiler” package in R. Additionally, Gene Set Enrichment Analysis (GSEA) was performed to identify the differences in the set of genes expressed between the two clusters in the enrichment of the KEGG pathway. The number of permutations was performed 1000 times for each analysis, and the pathways with adjusted *p* value  < 0.05 and q values  < 0.05 were considered statistically significant.

### Prediction of drug response

Based on the information retrieved from the Genomics of Drug Sensitivity in Cancer (GDSC) database (https://www.cancerrxgene.org/), the chemotherapeutic sensitivities for ccRCC were calculated. Three common clinical drugs were selected to predict the chemotherapeutic response, and the R package “pRRophetic” was used to estimate the chemotherapeutic response determined by the half-maximal inhibitory concentration (IC50).

### Cell lines and RT-qPCR analysis

The human ccRCC cell lines, 786-O and Caki-1, were provided by Haixiang Shen, and the normal human renal cortex epithelial cell line, HK-2, was provided by Yanhong Ma. RPMI 1640 medium (Gibco) supplemented with 10% fetal bovine serum (FBS, Gibco) was used to culture the cells. And all cells were cultured at 37 ℃ with 5% carbon dioxide. Total RNAs were extracted from the cells with Trizol Kit (Invitrogen, Carlsbad, CA, USA) according to the manufacturer’s instructions. The first strand (cDNA) was reverse-transcribed and used as the template for qPCR analysis as previously described [10]. The expression levels of pyroptosis regulators (AIM2, NOD2, GZMB, and CASP5) were measured using a SYBR Green qPCR Kit (Takara, Japan). The gene name and primer sequences are listed in Additional file 5: Table S2. The mRNA levels of all genes were normalized to GAPDH (an endogenous normalization reference).

### Statistical analysis

Statistical analysis was conducted using R software (http://www.R-project.org, version 4.0.3), PERL programming language (version 5.32.1.1, https://www.perl.org/), and GraphPad Prism 8.0. Correlation coefficients were computed by Spearman’s and distance correlation analyses. Kaplan–Meier curves were plotted, and a log-rank test was used to calculate the significant survival difference. The Wilcoxon signed-rank test was used to analyze continuous variables, whereas the chi-square test was used to analyze the categorical data. The RT-qPCR results were expressed as the mean  ±  SEM. The differences of groups were evaluated by one-way ANOVA followed by the ad hoc Dunnett’s multiple comparisons test to compare to normal kidney cells. A *p* value less than 0.05 was considered statistically significant for all analyses.

## Results

### Identification and classification of differentially pyroptosis-associated regulators

**Fig. 1:**
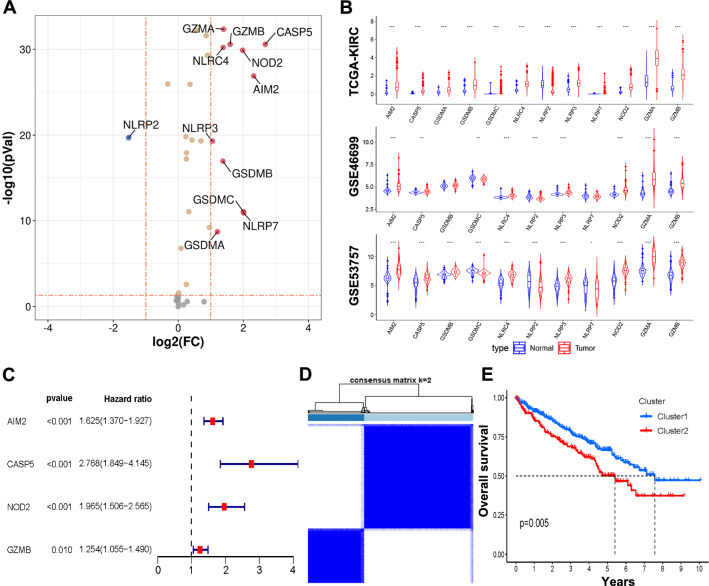
Identification and classification of pyroptosis regulators. **A** Volcano map and violin plots (**B**) of differential pyroptosis regulators expression in ccRCC tissue compared to normal tissues. **C** Univariate Cox regression analysis of pyroptosis regulators. **D** Consensus clustering matrix for k  = 2. **E** The Kaplan–Meier curves of the overall survival for the two clusters of ccRCC patients. *p  < 0.05, **p  < 0.01, and ***p  < 0.001

The expression of 37 pyroptosis-associated genes was compared in TCGA-KIRC cohort, and 11 genes were identified as differentially expressed genes (Fig. 1A). After two independent datasets from the GEO database were utilized to externally illustrate the expression of these 11 genes in ccRCC, six pyroptosis-associated regulators (AIM2, CASP5, NLRP3, NOD2, GZMA, and GZMB) demonstrated to be highly expressed in ccRCC (Fig. 1B). Combined with patient prognostic information downloaded from TCGA datasets, univariate Cox regression was then implemented to screen genes from these 6 regulators, and four of them (AIM2, CASP5, NOD2, and GZMB) were significantly related to ccRCC patient prognosis (Fig. 1C). To further investigate the relationship between the expression of these 4 regulators and ccRCC subtypes, consensus clustering analysis was performed with TCGA-KIRC cohort patients. The consensus clustering analysis was performed with ccRCC patients based on these four pyroptosis regulators, and the result showed that the clustering variable (k)  = 2 was identified as the highest clustering stability from k  = 1 to 9, indicating that ccRCC patients could be divided into two clusters with the highest intragroup correlations and the lowest intergroup correlations (Fig. 1D). Moreover, patients in Cluster 1 had favourable overall survival (OS) compared to those in Cluster 2 (Fig. 1E).

### TIME analysis of the clusters according to the pyroptosis regulators

**Fig. 2:**
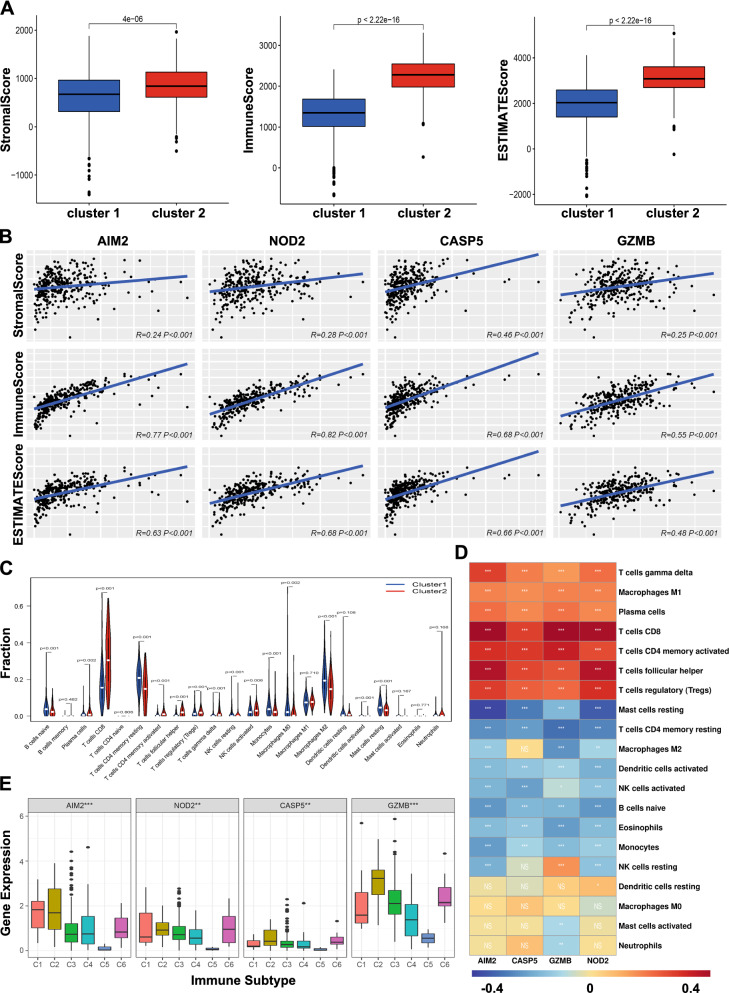
TIME characteristics among four pyroptosis regulators. **A** Different expression of ESTIMATE score, immune score, and stromal score between two clusters. **B** The correlation of ESTIMATE score, immune score, and stromal score with the four pyroptosis regulators. **C** Differences in the levels of infiltration of the immune cells in two clusters. **D** Heatmap of the correlation of the levels of infiltration of the immune cells with four pyroptosis regulators. **E** The correlation of pyroptosis regulators expression with immune infiltrate subtypes in ccRCC. *NS* no significant difference, *p  < 0.05, **p  < 0.01, and ***p  < 0.001

To investigate which pathways are enriched in the clusters, GSEA analysis was performed to identify the significant KEGG pathways associated with the two clusters. The results showed that cytokine receptor interaction, T cell receptor signaling pathway, antigen processing and presentation, chemokine signaling pathway, natural killer cell mediated cytotoxicity, toll like receptor signaling pathway, B cell receptor signaling pathway, and JAK STAT signaling pathway were significantly related to Cluster 2 (Additional file 1: Figure S1), indicating that the clusters containing pyroptosis regulators were associated with tumor immune microenvironment. Therefore, to explore the difference of immune function between the two clusters, ESTIMATE score were calculated and demonstrated that Cluster 2 had significantly higher ESTIMATE, stromal, and immune scores compared to Cluster 1 (Fig. 2A), and the four pyroptosis regulators were positively related with these scores (Fig. 2B). Furthermore, the two clusters revealed significant differences in immune cell infiltration (Fig. 2C). Cluster 1 possessed higher infiltrated levels of B cells naive, T cells CD4 memory resting, NK cells resting, monocytes, macrophages M0, macrophages M2, dendritic cells activated, and mast cells resting, while Cluster 2 was higher related with plasma cells, T cells CD8, T cells memory activated, T cells follicular helper, T cells regulatory (Tregs), T cells gamma delta, and NK cells activated. Analysis of the correlation between the four regulators and immune cell types showed that the four regulators had a positive or negative correlation with most immune cell types (Fig. 2D). Interestingly, further investigation on the differential expression of immune genes between two clusters revealed that a mass of immune genes was up-regulated in Cluster 2 while only 5 genes were down-regulated (Additional file 2: Figure S2A). The 10 most upregulated and downregulated genes were selected to analyze the correlation and the result are showed in Additional file 2: Figure S2B. GO analysis of biological processes showed that the differentially expression of immune genes between clusters was enriched in activating cell surface receptor signaling pathway and signal transduction of immune response. Cellular component analysis indicated that the differentially expressed immune genes were abundant in the immunoglobulin complex. Molecular function analysis indicated that the differentially expressed immune genes were involved antigen binding. KEGG analysis showed that the differentially expressed immune genes were enriched in cytokine-cytokine receptor interactions (Additional file 2: Figure S2C, D). These results indicated that Cluster 2 and the four regulators are implicated in the tumor immune microenvironment.

### Association of pyroptosis regulators with the immune checkpoint inhibitors and the targeted drug sensitivities

**Fig. 3:**
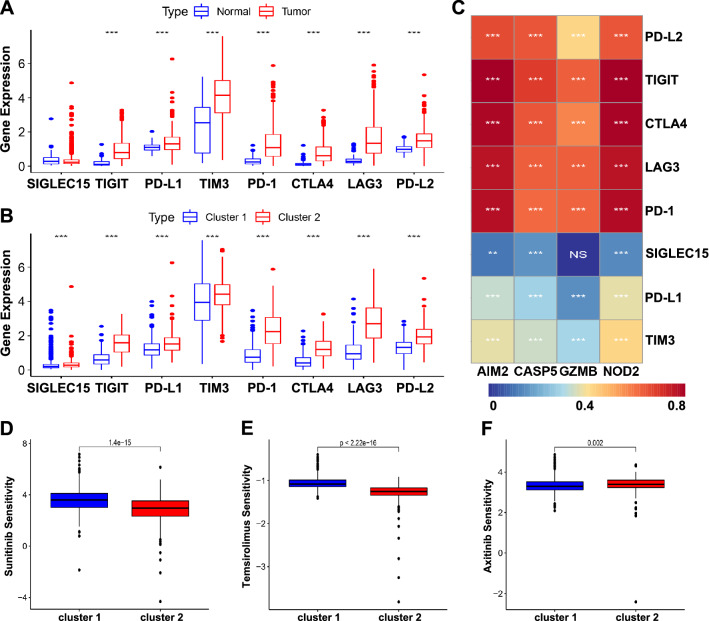
Association of pyroptosis regulators with the ICIs and the targeted drug sensitivities. **A** Box plots of differential ICIs expression between ccRCC and normal tissues. **B** Box plots of differential ICIs expression between two clusters. **C** Heatmap of the correlation of ICIs with four pyroptosis regulators. **D** Box plots of differentially targeted drug sensitivities between two clusters. *NS* no significant difference, *p  < 0.05, **p  < 0.01, and ***p  < 0.001

Immune checkpoint inhibitor (ICI) therapies have been established as a potent treatment option for multiple tumor types, while only a fraction of patients benefit from such therapy. Therefore, the differential expression of ICIs in different subtypes were further explored. Eight hot immune checkpoint genes (SIGLEC15, TIGIT, PD-L1, TIM3, PD-1, CTLA4, LAG3, and PD-L2) were positively related to Cluster 2 (Fig. 3A, B). Interestingly, four regulators were tightly related to the most ICIs, while there was no significant relation between GZMB and SIGLEC15 (Fig. 3C). Additionally, considering the clinical treatment value in the target drugs sensitivity, Sunitinib and Temsirolimus showed more sensitivity in Cluster 1, while Axitinib was closely related to Cluster 2 (Fig. 3D–F). These results indicated that this classification may provide a certain reference value for the clinical medication of different ccRCC patients.

### Stratification analysis of the cluster clinical utility

**Fig. 4:**
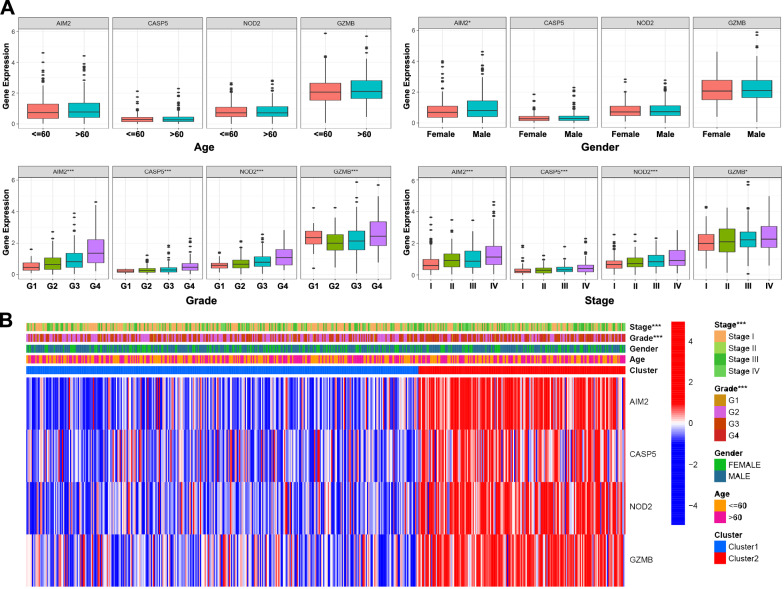
Stratification analysis of the cluster clinical utility. **A** Box plots of the four pyroptosis regulators expression between different clinical characteristics. **B** Heatmap of the correlation of the two clusters with ccRCC patient clinical characteristics

To further explore the clinical value of the relationship between this classification and different clinical characteristics, ccRCC patients were divided into different groups according to patient age, gender, grade, and disease stage. It is worth noting that the higher disease stage was more associated with the patients in Cluster 2, as well as the pathological grade (Table 1; Fig. 4B). Further analysis of the relationship between pyroptosis regulators and clinical features demonstrated that the expression level of the four regulators was risen with the increase of disease stage and pathological grade, while only AIM2 was more highly expressed in males than in the females. There was no significant difference between the expression of the four regulators and patient age (Fig. 4A).

**Table 1:** Clinical characteristics of two clusters of ccRCC patients

	Cluster 1 (n = 341)	Cluster 2 (n = 180)	p value
Age			
≤ 60	176 (51.6%)	85 (47.2%)	0.341
> 60	165 (48.4%)	95 (52.8%)	
Gender			
Female	122 (35.8%)	60 (33.3%	0.578
Male	219 (64.2%)	120 (66.7%)	
Stage			
I	192 (56.3%)	69 (38.3%)	< 0.001
II	31 (9.1%)	23 (12.8%)	
III	76 (22.3%)	47 (26.1%)	
IV	42 (12.3%)	41 (22.8%)	
Grade			
G1	10 (2.9%)	4 (2.2%)	< 0.001
G2	169 (49.6%)	57 (31.7%)	
G3	132 (38.7%)	74 (41.1%)	
G4	30 (8.8%)	45 (25%)

### Experimental verification of the four regulators expression

**Fig. 5:**
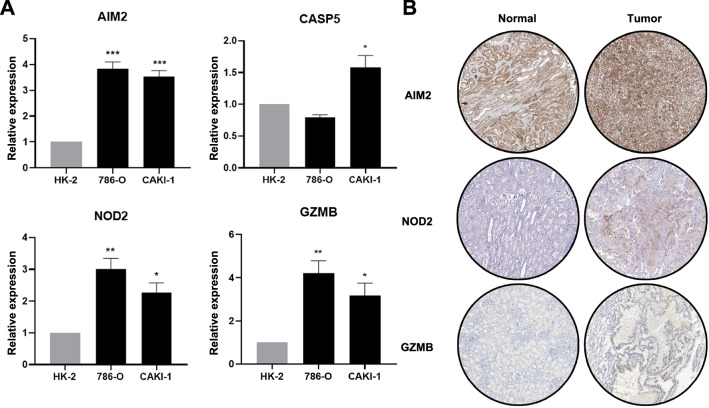
Verification of the four regulators expression. **A** Four pyroptosis regulators gene expression in normal and ccRCC cell lines. **B** The immunohistochemistry staining of three pyroptosis in normal kidney tissue and renal cancer tissue were shown from the HPA database (HPA Antibody ID: HPA031365, HPA003418, HPA041985;  × 10)

Based on the bioinformatic analysis of the expression of these four regulators, we analysed the mRNA and protein expression levels. Compared to normal cells, the expression levels of these 4 genes were upregulated to varying degrees in the two ccRCC cell lines (Fig. 5A). Correspondingly, the protein expression patterns of AIM2, NOD2, and GZMB from the HPA database revealed that the regulators were also overexpressed in ccRCC tissue compared to normal tissue (Fig. 5B).

### Four pyroptosis regulator expression patterns in multiple cancers

**Fig. 6:**
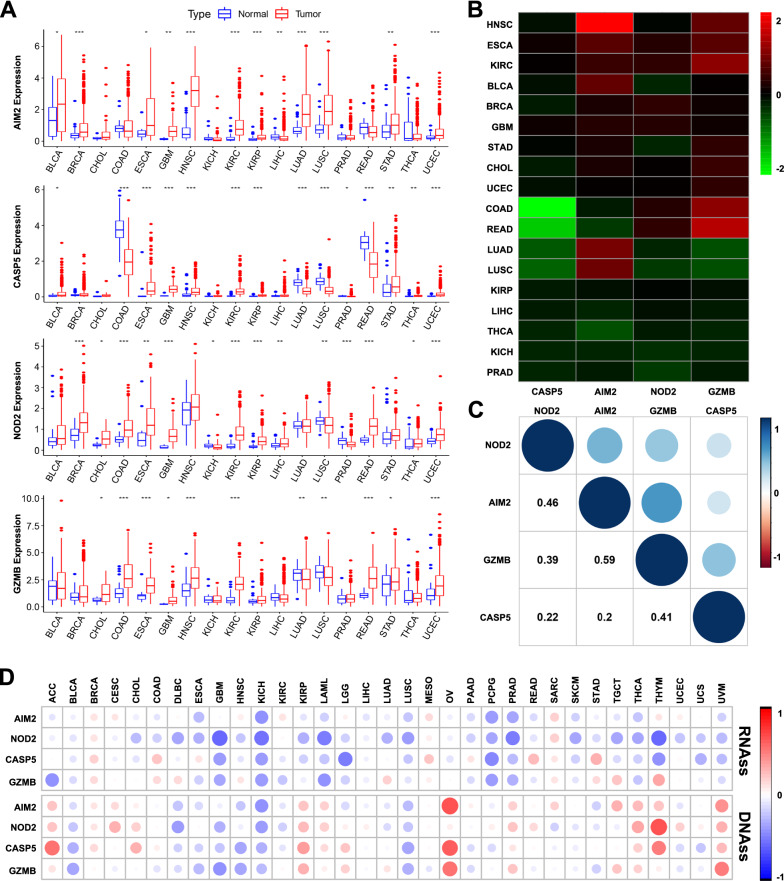
Pan-cancer analysis of the four pyroptosis regulators expression pattern. **A** Four pyroptosis regulators expression levels in different cancer types and normal tissues. **B** Four pyroptosis regulators expression levels in different cancer types from TCGA data. **C** Correlation between the four pyroptosis regulators. **D** Four pyroptosis regulators expression associated with RNAss and DNAss in different cancers

Further investigation of the expression pattern of these four pyroptosis regulators in pan-cancer showed that four pyroptosis regulators were differentially expressed in multiple cancers (Fig. 6A). AIM2 was differentially expressed in multiple cancers, including bladder, breast, esophageal, kidney, liver, lung, stomach, and endometrium, as well as CASP5, NOD2, and GZMB (Fig. 6A). Further analysis found that AIM2 was the most positively related to HNSC, while CASP5 was negatively related with COAD (Fig. 6B). And AIM2 and GZMB were the two genes with the most significant positive association (Correlation coefficient  = 0.59, Fig. 6C).

During cancer progression, tumor cells can gradually lose a differentiated phenotype and acquire progenitor and stem-cell-like features [11]. Generally, the RNAss and the DNAss can be used to assess the stemness of cancer stem cells [12]. Therefore, the correlation between pyroptosis regulators with tumor stemness measured by RNAss and DNAss was explored. The four regulators were significantly positively or negatively correlated with RNAss and DNAss in most of the pan-cancers (Fig. 6D). Notably, in KIRC, AIM2 and GZMB were positively or negatively associated with RNAss, respectively, while NOD2 was negatively associated with DNAss (Additional file 3: Figure S3).

### Association of the four pyroptosis regulators with tumor immune microenvironment in pan-cancer

**Fig. 7:**
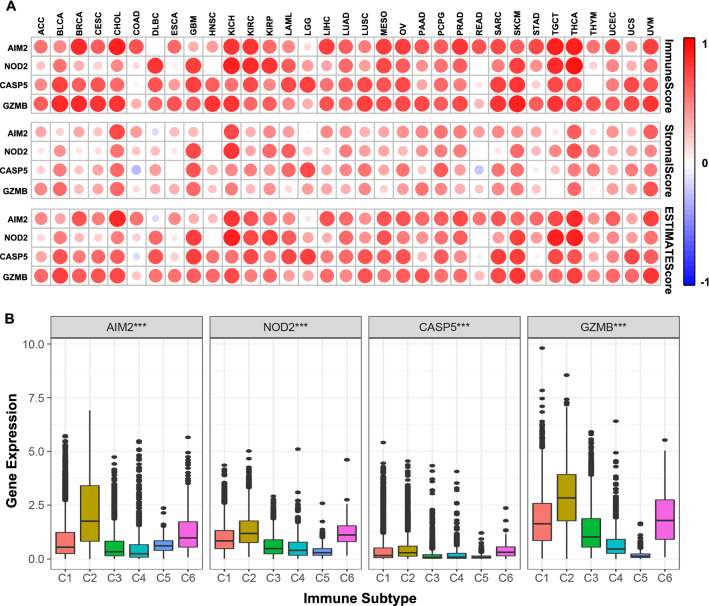
Pan-cancer TIME analysis of the four pyroptosis regulators. **A** Associations of the four pyroptosis regulators expression with stromal score, immune score, and ESTIMATE score in multiple cancers. **B** The correlation of pyroptosis regulators expression with immune infiltrate subtypes in multiple cancers. *C1* wound healing, *C2* INF-r dominant, *C3* inflammatory, *C4* lymphocyte depleted, *C5* immunologically quiet, *C6* TGF-β dominant. ***p  < 0.001

Based on the important regulatory effect of pyroptosis genes on different immune cell types, the four regulators were further investigated in the pan-cancer microenvironment. Interestingly, except AIM2 was negatively associated with DLBC and CASP5 was negatively associated with COAD and READ in ESTIMATE score and stromal score, others were positively associated with cancers in the immune score, stromal score, and ESTIMATE score (Fig. 7A). To explore the four pyroptosis regulators expression in different immune subtypes in pan-cancer, Immune infiltrates were divided into six types for analysis which correspond from tumor-promoting to tumor suppressive, respectively, including wound healing, IFN-γ dominant, inflammatory, lymphocyte depleted, immunologically quiet, and TGF-β dominant. Interestingly, the four pyroptosis regulators were all significantly related to immune subtypes, and the four pyroptosis regulators were most highly expressed in IFN-γ dominant, followed by TGF-β dominant (Fig. 7B). In KIRC, however, the expression of the four pyroptosis regulators was the highest in C2 as same as pan-cancer analysis. AIM2, which had the second-highest expression in immune subtypes, was related with C1, while NOD2, CASP5, and GZMB were related to C6 (Fig. 2E).

## Discussion

Surgical resection is the first treatment for renal cell carcinoma in our clinical practice. However, it is still difficult to remove renal cell carcinoma metastasis. Metastatic renal cell carcinoma is resistant to radiotherapy and systemic therapy, including hormone therapy, and chemotherapy [13]. A clinical trial of nivolumab, an anti–PD-L1 immune checkpoint inhibitor, for the treatment of metastatic ccRCC (CheckMate-025) has demonstrated that nivolumab has significantly superior OS, hazard ratio, ORR and overall response rate [14]. These studies indicate that the application of immunotherapy in the treatment of ccRCC has great prospects. An in-depth analysis of the heterogeneity of the ccRCC TIME may help to provide precise and individualized immunotherapy management to improve the treatment effect and the quality of the patient's prognosis survival. Here, ccRCC patients were separated into two independent molecular subtypes with differential clinical characteristics, immune gene expression, and TIME through consensus clustering for four main pyroptosis regulators. According to the present study, Cluster 2 had a worse prognosis than Cluster 1, but Cluster 2 was more sensitive to immunotherapy.

Among the intrinsic death mechanisms of cells, pyroptosis has received increasing attention. Pyroptosis is a newly recognized type of programmed inflammatory cell death that is activated through pathways mediated by classic caspase-1 inflammasomes or non-classical caspase-4, caspase-5 and caspase-11 [15]. Recently, emerging evidence has reported the important role of pyroptosis in the tumorigenesis of human malignancies [16, 17]. Pyroptosis inhibits tumour growth in colorectal cancer, liver cancer, and skin cancer, but it has a two-way effect on breast cancer [18–20]. In ccRCC, it remains unknown how pyroptosis-related genes interact and whether they are related to patient survival time. The expression of most pyroptosis regulatory genes is positively correlated and plays an important prognostic role in ccRCC. These findings suggest that pyroptosis regulators may play a key role in regulating tumorigenesis and ccRCC development. In the present study, four pyroptosis regulators that are highly expressed in kidney cancer and cause poor prognosis were identified. AIM2 is a cytosolic innate immune receptor that recognizes double-stranded DNA (dsDNA) released during cellular perturbation and pathogenic assault. Activated AIM2 activates the classical pathway of pyroptosis through caspase-1 [21]. AIM2 may play a unique role in different cancer types [22–25], and present study showed that AIM2 plays a cancer-promoting role in ccRCC. Caspase-5 (CASP5), similar to caspase-4 and caspase-11, is an executive protein of caspase-1-independent pyroptosis [26]. GZMB is used by cytotoxic lymphocytes as a molecular weapon for defence against virus-infected and malignantly transformed host cells [27]. The low expression of GZMB is related to early metastasis in colorectal cancer, suggesting the infiltration of blood vessels and nerves [28]. Interestingly, our analysis found that GZMB is a positively correlated gene in kidney cancer. NOD2 is a pattern recognition receptor that can regulate the host's innate immune response and prevent inflammation, steatosis and obesity [29]. Extracellular histone H3 induced by LPS could cause pyroptosis during sepsis via NOD2 and VSIG4/NLRP3 pathway [30]. It remains unknown how these genes interact with each other during cell pyroptosis in ccRCC.

In our clinical practice, vascular endothelial growth factor receptor tyrosine kinase inhibitors (VEGFR-TKIs) have become the standard of care for mRCC [31]. Sunitinib, a broad-spectrum inhibitor of receptor tyrosine kinases, has been widely used as the standard of treatment for first-line therapy of advanced ccRCC [32]. However, not all RCC patients are sensitive to sunitinib treatment, and most patients will develop resistance to sunitinib after a few months of treatment. Therefore, the key to selecting sunitinib for treatment is to selecte patients who are sensitive to sunitinib. Present study demonstrated that Cluster 1 is more sensitive to sunitinib, while Cluster 2 is more sensitive to axitinib. Understanding the expression of pyroptosis regulators in ccRCC before treatment may help to design more optimized targeted treatment options to benefit patients.

Some studies have shown that the effective proinflammatory effect of pyroptosis is related to the regulation of tumor immune microenvironment [33]. However, the potential role of pyroptosis in the immune microenvironment of ccRCC is still elusive. Based on the expression characteristics of pyroptosis regulators, the present study indicated that patients in different clusters have different correlations with different immune infiltrations. Higher infiltrated levels of B cells naive, T cells CD4 memory resting, NK cells resting, monocytes, macrophages M0, macrophages M2 were observed in Cluster 1. On the other hand, Cluster 2 was higher related with CD8  +  T cell, Tregs, and T follicular helper cells. Tregs can secrete a variety of immunosuppressive cytokines, leading to immune escape of tumor cells. Studies have shown that Tregs effectively inhibit the proliferation of effector T cells and a high CD8  +  T cell infiltration level is an unfavorable prognostic factor for ccRCC [34, 35]. The poor prognosis of Cluster 2 and the conclusions of these two conclusions are mutually consistent. Cluster 2 is positively correlated with eight hot ICIs (SIGLEC15, TIGIT, PD-L1, TIM3, PD-1, CTLA4, LAG3, and PD-L2). Based on these findings, the poor survival results of Clusters 2 may be caused by the reduced level of anti-tumor immunity, and Cluster 2 may be more beneficial in immunotherapy. The results of GSEA indicate that Cluster 2 is related to a variety of immune regulatory signaling pathways. Importantly, the JAK-STAT signaling pathway is related to the carcinogenesis and immune infiltration of ccRCC. Thus, we speculated that pyroptosis may regulate the composition of the tumor immune microenvironment. Our study suggested that ccRCC with high expression of these 4 genes may be more sensitive to immunotherapy.

The expression level of the four regulators increased with increasing disease stage and pathological grade, and there was no significant difference between the expression of the four regulators and patient age, which was consistent with the worse prognosis of Cluster 2.

Given that pyroptosis is widely involved in various biological processes, we investigated whether these four pyroptosis regulators in Cluster 2 also play an important role in other cancer types. A pan-cancer analysis was subsequently performed, and the results showed that these four pyroptosis regulators were closely related to the stromal score, immune score, and ESTIMATE score in multiple cancer types. These results indicated that these four pyroptosis regulators are potential targets for a variety of cancer immunotherapies and not limited to ccRCC.

In the future, further reliable verification analysis is needed in a larger cohort. In addition, additional in vitro and in vivo experiments are needed to clarify the regulatory mechanism between pyroptosis regulators and tumor immune microenvironment.

## Conclusions

In summary, this study systematically analyzed the expression profile of pyroptosis regulators in ccRCC and its correlation with immune checkpoints and the role in the tumor immune microenvironment. The expression of pyroptosis regulators is significantly related to immune checkpoints such as PD-L1 and CTLA4. Two independent subtypes were also established through the consistent clustering of pyroptosis regulators. Tumour heterogeneity and differences in immune checkpoint expression and tumor immune microenvironment were observed between the two subtypes, which will help risk stratification and precise treatment of ccRCC patients. A one-step pan-cancer analysis showed that these pyroptosis regulators are closely related to stromal score, immune score, and ESTIMATE score in many cancer types. Importantly, future clinical trials and basic research will help determine targets for improving the efficacy of cancer immunotherapy.

## Supplementary Information

**Additional file 1: Figure S1.** Gene set enrichment analysis (GSEA) indicating that tumor hallmarks are enriched in the Cluster 2.

**Additional file 2: Figure S2.** Immune gene analysis between the two clusters. **A** Volcano map of differential pyroptosis regulators expression in ccRCC compared to normal tissues. **B** Association of 10 upregulated and 5 downregulated immune genes between the two clusters. GO **C** and KEGG **D** analysis of differentially expressed immune genes between the two clusters.

**Additional file 3: Figure S3.** Correlation of the four pyroptosis regulators expression associated with RNAss and DNAss in ccRCC.

**Additional file 4: Table S1.** The list of pyroptosis related genes.

**Additional file 5:** **Table S2.** Primer information.

## Data Availability

The datasets exhibited in present study can be discovered in online repositories. The names of the repository/repositories and accession number(s) can be found in the article/Additional file.
