# Supplementary figures and images for: Comprehensive analysis of pyroptosis regulators and tumor immune microenvironment in clear cell renal cell carcinoma
Source: Cancer Cell Int. 2021 Dec 14;21:667. doi: 10.1186/s12935-021-02384-y (PMC8670029; doi:10.1186/s12935-021-02384-y)

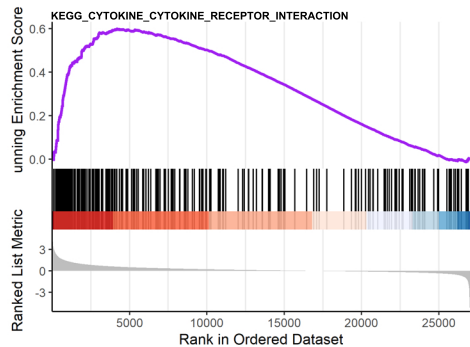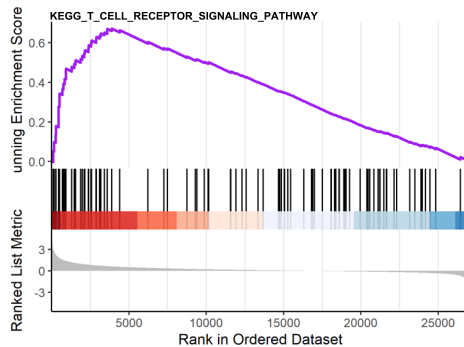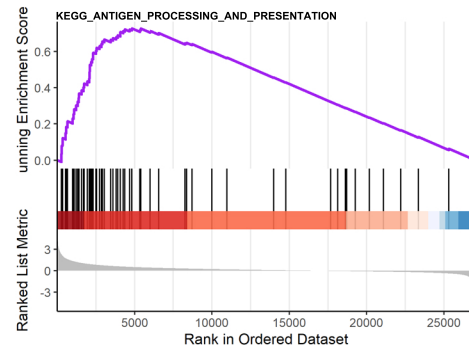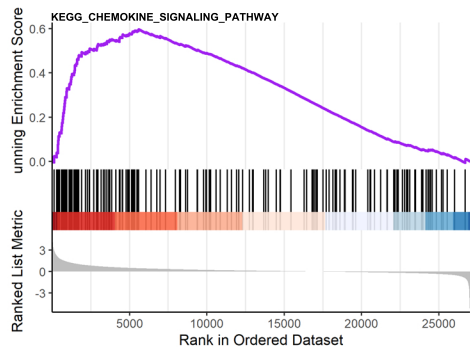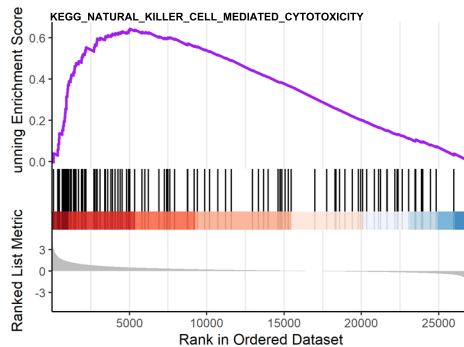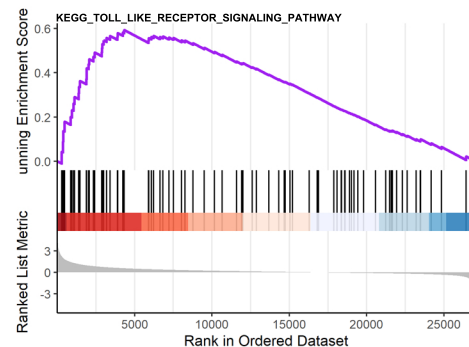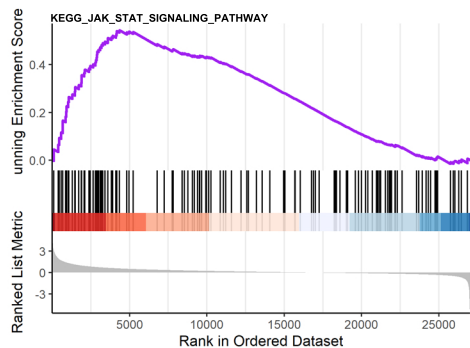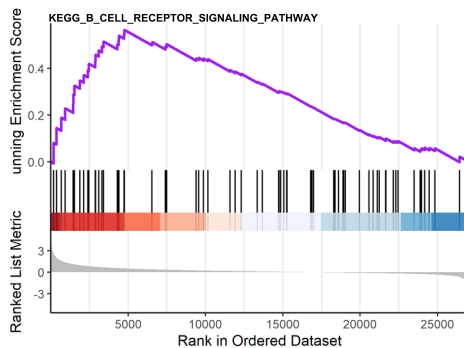

Supplement: Supplementary file 1 — Additional file 1: Figure S1. Gene set enrichment analysis (GSEA) indicating that tumor hallmarks are enriched in the Cluster 2. [file 12935_2021_2384_MOESM1_ESM.pdf]

**A**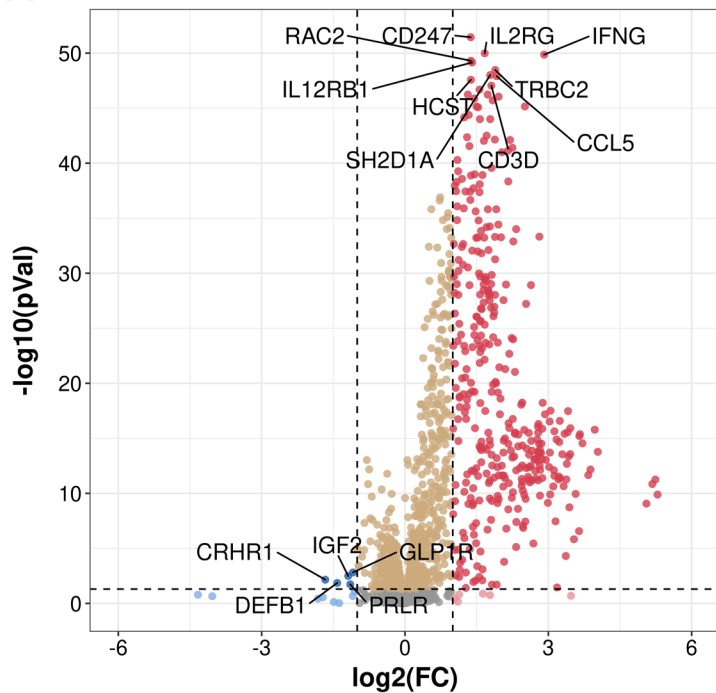**B**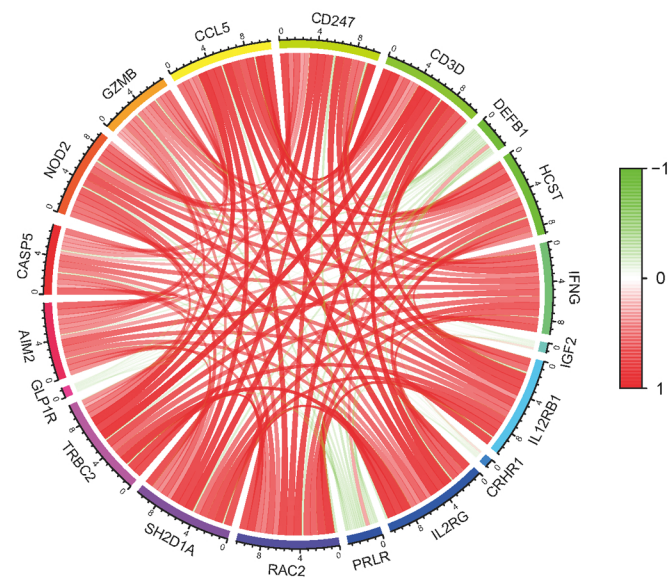**C**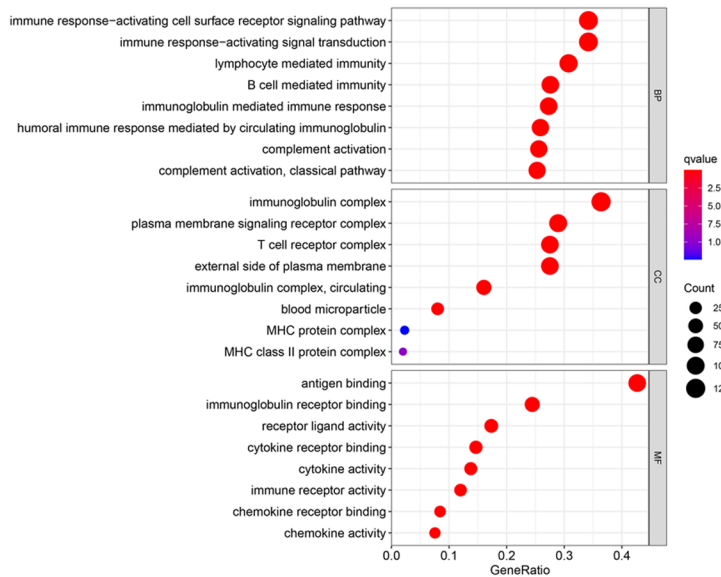**D**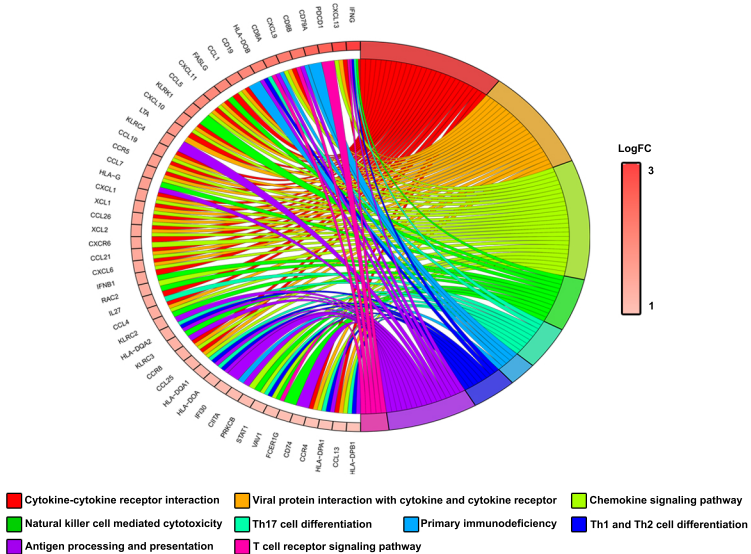

Supplement: Supplementary file 2 — Additional file 2: Figure S2. Immune gene analysis between the two clusters. A Volcano map of differential pyroptosis regulators expression in ccRCC compared to normal tissues. B Association of 10 upregulated and 5 downregulated immune genes between the two clusters. GO C and KEGG D analysis of differentially expressed immune genes between the two clusters. [file 12935_2021_2384_MOESM2_ESM.pdf]

AIM2

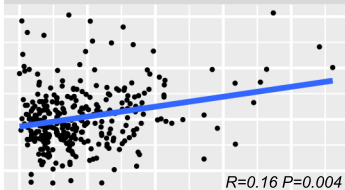

NOD2

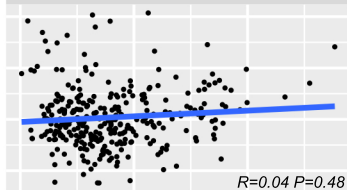

CASP5

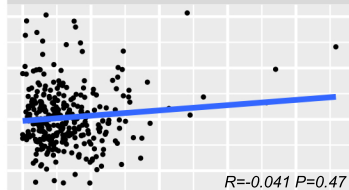

GZMB

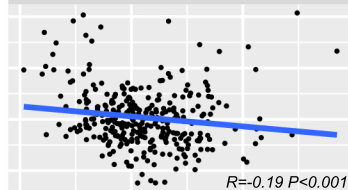

RNASS

 $R=-0.083$   $P=0.14$ 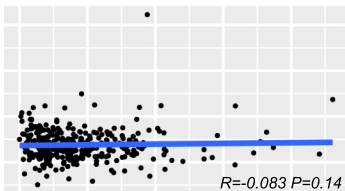 $R=-0.18$   $P=0.0018$ 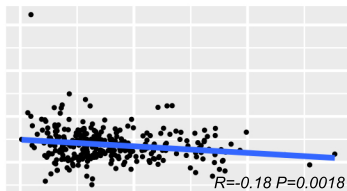 $R=-0.093$   $P=0.1$ 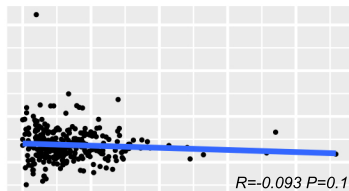 $R=0.026$   $P=0.65$ 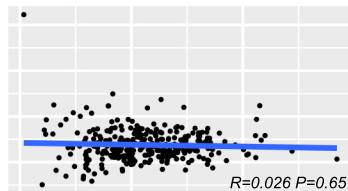

DNASS

Gene Expression

Supplement: Supplementary file 3 — Additional file 3: Figure S3. Correlation of the four pyroptosis regulators expression associated with RNAss and DNAss in ccRCC. [file 12935_2021_2384_MOESM3_ESM.pdf]
