# Supplementary material for: Comprehensive analysis of pyroptosis regulators and tumor immune microenvironment in clear cell renal cell carcinoma
Source: Cancer Cell Int. 2021 Dec 14;21:667. doi: 10.1186/s12935-021-02384-y (PMC8670029; doi:10.1186/s12935-021-02384-y)
Supplement: Supplementary file 5 — Additional file 5: Table S2. Primer information. [file 12935_2021_2384_MOESM5_ESM.docx]

**Supplementary Table S2. Primer information**

| **Primer**  **Symbol** | **Gene name** | **Primer direction** | **Sequences (5’to 3’)** | **PCR**  **(bp)** | **Accession** |
| --- | --- | --- | --- | --- | --- |
| *NOD2* | Nucleotide binding oligomerization domain containing 2 | Forward | CGCACCGAGTTCAACCTCAAGG | 149 | NM_001370466.1 |
|  |  | Reverse | AAGACAGGCAGGTGGCACAAAC |  |  |
| *GZMB* | Granzyme B | Forward | AGGTGCGGTGGCTTCCTGATAC | 117 | NM_001346011.2 |
|  |  | Reverse | CTGGGTCGGCTCCTGTTCTTTG |  |  |
| *AIM2* | Absent in melanoma 2 | Forward | AAAGCTGGTGAAACCCCGAA | 168 | NM_001348247.2 |
|  |  | Reverse | GTCCTCGTTTCTAACCCCCA |  |  |
| *CASP5* | Cysteine-aspartic acid protease-5 | Forward | AGATGTTGGAATACCTGGGCAA | 461 | NM_001136112.3 |
|  |  | Reverse | AAGCAGCCTTTTCATCCCCA |  |  |
| *GAPDH* | glyceraldehyde-3-phosphate dehydrogenase | Forward | GTCAAGGCTGAGAACGGGAA | 158 | NM_001256799.3 |
|  |  | Reverse | AAATGAGCCCCAGCCTTCTC |  |  |
|  |  |  |  |  |  |
